# A Risk-Oriented and Explainable Hierarchical AI Framework for Chronic Kidney Disease Classification

**DOI:** 10.3390/diagnostics16081157

**Published:** 2026-04-14

**Authors:** Sara Alhaifi, Fatmah M. A. Naemi, Nahed Alowidi

**Affiliations:** 1 Department of Computer Science, Faculty of Computing and Information Technology, King Abdulaziz University, Jeddah 21589, Saudi Arabia; nalowidi@kau.edu.sa; 2 Histocompatibility and Immunogenetics Laboratory, King Fahad General Hospital, Jeddah 21589, Saudi Arabia; fnaemi@moh.gov.sa

**Keywords:** chronic kidney disease, machine learning, hierarchical classification, risk prediction, explainable artificial intelligence

## Abstract

**Background/Objectives:** Chronic kidney disease (CKD) remains a major public health challenge due to its silent progression and late clinical detection. Recent advances in machine learning have demonstrated promising performance in CKD detection; however, most existing approaches focus primarily on binary classification or rely on longitudinal or specialized biomarkers that are not routinely available in clinical practice. While several studies attempt risk stratification, few integrate risk modeling with stage-aware hierarchical decision frameworks suitable for routine clinical workflows. This study proposes a risk-oriented, explainable, and hierarchical machine learning framework for CKD classification using real-world laboratory data from 746 patients in a Saudi population. **Methods:** The proposed framework is designed as a hierarchical machine learning pipeline that mirrors clinical practice by sequentially identifying CKD presence, performing disease staging only for confirmed cases, and estimating risk for individuals without overt CKD. Specifically, an XGBoost model with recursive feature elimination (RFE) was employed for binary CKD detection, followed by a multilayer perceptron (MLP) model with SelectKBest for stage classification. A unified preprocessing pipeline, clinically informed feature selection, and validated machine learning models were employed to develop the hierarchical prediction system. **Results:** The system achieved 97% accuracy and F1-score in binary CKD classification, and up to 85% accuracy and 86% F1-score in stage classification. In addition, an interpretable risk scoring mechanism and SHAP-based explanations enabled early identification of CKD-like laboratory patterns using routine laboratory tests. **Conclusions:** The proposed system provides a transparent and deployable framework that supports preventive nephrology and clinically meaningful decision-making.

## 1. Introduction

Chronic kidney disease (CKD) is a major global public health concern associated with substantial morbidity, mortality, and economic burden [1,2,3]. Clinically, CKD is conventionally classified into five stages (stages 1–5), reflecting progressively worsening kidney function. In stages 1 and 2, patients are often asymptomatic, and kidney function, as reflected by the estimated glomerular filtration rate (eGFR), may remain within or near normal ranges, making early detection using routine laboratory tests particularly challenging [4,5]. Instead, diagnosis at these stages relies primarily on the identification of persistent markers of kidney damage—most notably albuminuria—typically assessed using the urine albumin-to-creatinine ratio (UACR) or timed urine protein measurements over a minimum period of three months [6,7]. Such assessments are not routinely included in standard laboratory panels for population-level screening or general health evaluations and are typically reserved for targeted testing in high-risk individuals or for confirmatory diagnosis, often requiring longitudinal follow-up [8]. In contrast, advanced CKD stages are characterized by sustained reductions in eGFR and more pronounced biochemical abnormalities that are readily captured by routine blood tests and are often accompanied by clinical symptoms [9]. While this facilitates diagnosis, detection at advanced stages usually indicates substantial and largely irreversible kidney damage. Consequently, early identification of CKD remains essential, as timely intervention can slow disease progression, reduce complications, and improve long-term outcomes [10,11].

Recognizing individuals at elevated risk before the onset of advanced disease represents a key opportunity for preventive care [12]. In recent years, data-driven risk assessment systems have emerged as valuable tools in medical decision-making, enabling early prediction of disease susceptibility by integrating complex patterns across clinical and laboratory data [13,14]. Within the CKD domain, existing studies have largely focused on binary disease classification—distinguishing CKD from non-CKD—or on early prediction using AI-based models trained on longitudinal data collected over extended periods. While such approaches demonstrate strong predictive performance, they often rely on specialized biomarkers such as albuminuria, repeated measurements over time, or data modalities that are not routinely available in everyday clinical practice [15,16]. This gap between methodological sophistication and real-world feasibility poses a practical challenge. In routine care settings, laboratory testing for many individuals is limited to standard blood and urine panels that do not include the sensitive markers required for early-stage CKD diagnosis [7]. Consequently, there is a need for predictive systems that align with routine clinical workflows, particularly the standard outpatient evaluation process in which decisions are based primarily on commonly ordered laboratory panels without mandatory specialized biomarkers or longitudinal follow-up.

Motivated by this challenge, the present study proposes a clinically grounded prediction framework designed to operate within the constraints of routine laboratory testing. Rather than attempting to directly detect early-stage CKD using markers that are often unavailable, the proposed system adopts a hierarchical strategy that prioritizes reliable predictions where routine data are most informative. The model first identifies individuals with advanced CKD (stages 3–5), followed by disease staging for confirmed cases. For individuals classified as non-CKD or potentially in early stages, the framework shifts focus from diagnosis to risk assessment by analyzing routine laboratory profiles and quantifying their similarity to patterns observed in CKD patients. Based on this analysis, a continuous risk score is generated, and laboratory features contributing to elevated risk are highlighted for clinical review. This design supports targeted decision-making by indicating when further specialized testing or closer monitoring may be warranted. The results of this study support the feasibility of a clinically aligned, hierarchical strategy that leverages routinely available laboratory data to (i) reliably identify advanced CKD and estimate disease severity among confirmed cases, and (ii) provide an interpretable risk assessment for individuals without a definitive CKD diagnosis, guiding when further targeted testing or closer monitoring may be warranted.

The main contributions of this study can be summarized as follows:

1. A hierarchical CKD classification framework is developed and validated to separate advanced disease detection from severity staging and individualized risk assessment, closely aligning the modeling strategy with real-world clinical workflows.
2. A continuous risk scoring mechanism is designed for individuals without confirmed CKD, leveraging routinely collected laboratory profiles to enable early risk stratification and targeted clinical follow-up.
3. A real-world clinical laboratory dataset from kidney clinics in Saudi Arabia is curated and analyzed, addressing the scarcity of region-specific evidence in CKD prediction research.
4. Feature selection and SHAP-based explainability are integrated within the predictive pipeline to deliver transparent, interpretable, and clinically meaningful decision support.

The remainder of this paper is organized as follows. Section 2 reviews recent related work on CKD prediction, highlighting current approaches and their limitations. Section 3 describes the materials and methods, including dataset construction, preprocessing, feature selection, and hierarchical model development. Section 4 presents experimental results. Section 5 discusses the findings presented in Section 4. Section 6 introduces the proposed CKD risk assessment system and details its design, deployment workflow, and explainability components. Finally, Section 7 concludes the paper and outlines directions for future research.

## 2. Related Work

The prediction of chronic kidney disease (CKD) using laboratory and clinical data has gained substantial attention in recent years, driven by the need for early diagnosis and improved clinical decision support. Most existing studies employ data-driven modeling approaches to identify CKD based on key biomarkers such as serum creatinine, glomerular filtration rate (GFR), and albuminuria, with the primary objective of enabling early detection and risk-aware intervention. To ensure methodological relevance and quality, this review focuses on peer-reviewed journal and conference studies published between 2023 and 2025, resulting in a curated set of nine highly relevant works.

A subset of recent studies emphasizes early CKD detection using real-world or population-based clinical datasets, aiming to improve generalizability beyond benchmark datasets. He et al. [17] developed an explainable early-stage CKD prediction model using multicenter clinical data collected from three hospitals in China. The task was formulated as binary classification (early CKD vs. non-CKD) based on tabular demographic information, blood routine tests, urinalysis, and biochemical markers. Among the evaluated models, XGBoost achieved the best performance and demonstrated robustness through both internal and external validation. Model interpretability was provided using SHAP, which identified proteinuria and age as dominant predictors. Importantly, the authors deployed the model as a web-based application, highlighting its potential for real-world clinical screening. Notably, the model outputs were further translated into probabilistic estimates indicating the likelihood of early-stage CKD, enabling risk-aware screening at the individual level. Similarly, Bahrami et al. [18] developed binary CKD prediction models using large-scale tabular data from the Mashhad Stroke and Heart Atherosclerotic Disorders (MASHAD) population-based cohort. The models integrated sociodemographic, anthropometric, and laboratory features and employed Random Forest and feedforward neural networks, with the Random Forest model for women achieving the highest performance (AUC up to 0.90). A notable contribution of this work is the gender-specific modeling strategy, which revealed distinct risk patterns between men and women. Model transparency was supported through post hoc explainability using LIME, along with feature importance and partial dependence analyses.

In contrast to studies based on real-world or population-level clinical data, a substantial body of the literature relies on publicly available benchmark datasets, most notably the UCI CKD dataset [19]. Within this context, Kalpana et al. [20] proposed a stacking-based explainable boosting framework for binary CKD detection, in which multiple base learners were integrated using an Explainable Boosting Classifier (EBC) as a meta-learner. Owing to the intrinsic interpretability of the Generalized Additive Model (GAM) structure, the proposed model provided transparent feature-level explanations while achieving perfect evaluation metrics on the dataset. Building on similar benchmark-driven settings, Halder et al. [21] introduced ML-CKDP, a comprehensive binary classification framework that emphasized extensive preprocessing, multiple feature selection strategies, and systematic evaluation across different train–test split ratios and cross-validation schemes. Across all experimental configurations, Random Forest and AdaBoost consistently achieved the best performance, reporting near-perfect accuracy and 100% AUC. The study further demonstrated practical applicability through the development of a real-time web-based prediction system. Further extending this line of work, Islam et al. [4] investigated CKD prediction using the same dataset by applying Recursive Feature Elimination to derive a compact subset of 11 features from an initial pool of 25. After comparing multiple classifiers, XGBoost combined with PCA achieved the highest predictive performance, with accuracy approaching 0.99. Correlation analysis revealed strong associations between CKD and well-established clinical markers, including blood pressure, albumin, serum creatinine, and blood urea nitrogen, reinforcing their relevance in CKD prediction. Similarly, Surekha et al. [22] evaluated several classification models on the UCI CKD dataset and reported that a soft Voting Classifier achieved the best average performance (accuracy ≈ 98%). To improve model transparency, SHAP was employed to provide both global and local explanations of feature contributions. More recently, Alsekait et al. [23] proposed a stacked ensemble framework that combined recurrent deep architectures (RNN, LSTM, and GRU) with an SVM meta-learner, while systematically assessing multiple feature selection techniques. The best-performing configuration, based on mutual information-selected features, achieved an accuracy of 99.69%, with post hoc explainability supported through LIME. Despite the consistently high predictive performance reported across these benchmark-based studies, their heavy reliance on small and homogeneous datasets raises concerns regarding potential overfitting and limits confidence in the external validity and clinical generalizability of the proposed models when applied to real-world patient populations.

Moving beyond disease presence, a smaller subset of studies has explored prediction tasks that reflect CKD severity or progression. Lu et al. [24] proposed a progression-oriented binary prediction framework that distinguishes mild from advanced CKD based on 24 h urine protein levels. Unlike early detection studies, all participants in this work were CKD patients, and the task focused on disease severity rather than diagnosis. Using tabular clinical data from 1358 patients, the authors reduced an initial set of 100 variables to 17 key features using recursive feature elimination with logistic regression. Logistic regression achieved the best single-model performance (AUC = 0.85), while a soft-voting ensemble further improved performance (AUC = 0.856). Post hoc explainability using SHAP identified vitamin D, albumin, cystatin C, and transferrin as key predictors. Although the formulation remains binary, this study represents an intermediate step between early detection and full-stage modeling, providing clinically meaningful insights into CKD progression.

Beyond snapshot-based laboratory data, relatively few studies have explored longitudinal data to support earlier, risk-oriented CKD prediction. Saif et al. [25] proposed Deep-Kidney, a framework designed to predict CKD occurrence 6 and 12 months in advance using large-scale longitudinal data from Taiwan’s National Health Insurance Research Database. The study evaluated several deep architectures, with an ensemble model achieving the best performance (accuracy ≈ 0.993 for 6-month and 0.992 for 12-month prediction). While effective at a population level, the approach does not explicitly model clinical disease stages or rely on detailed laboratory validation for individual patient diagnosis.

Overall, the reviewed literature demonstrates the effectiveness of data-driven approaches for CKD prediction, particularly for early detection using laboratory and clinical data. However, several limitations remain evident. Most studies formulate CKD prediction as a binary task, treating the disease as a static condition and overlooking severity stratification or progression. Additionally, many high-performing models rely on public benchmark datasets with limited external validation, raising concerns about generalizability. Although explainability techniques such as SHAP and LIME are increasingly adopted, they are often applied post hoc rather than being systematically integrated into clinically aligned prediction frameworks. These gaps motivate the development of interpretable, real-world models that move beyond binary detection toward progression-aware and hierarchical CKD characterization, more closely reflecting clinical decision-making processes.

## 3. Materials and Methods

This section outlines the design and experimental setup of the proposed CKD classification framework. It presents the system overview, dataset description, preprocessing procedures, feature selection strategies, and model development process, including the hierarchical training configuration.

### 3.1. Proposed System Overview

**Figure 1:**
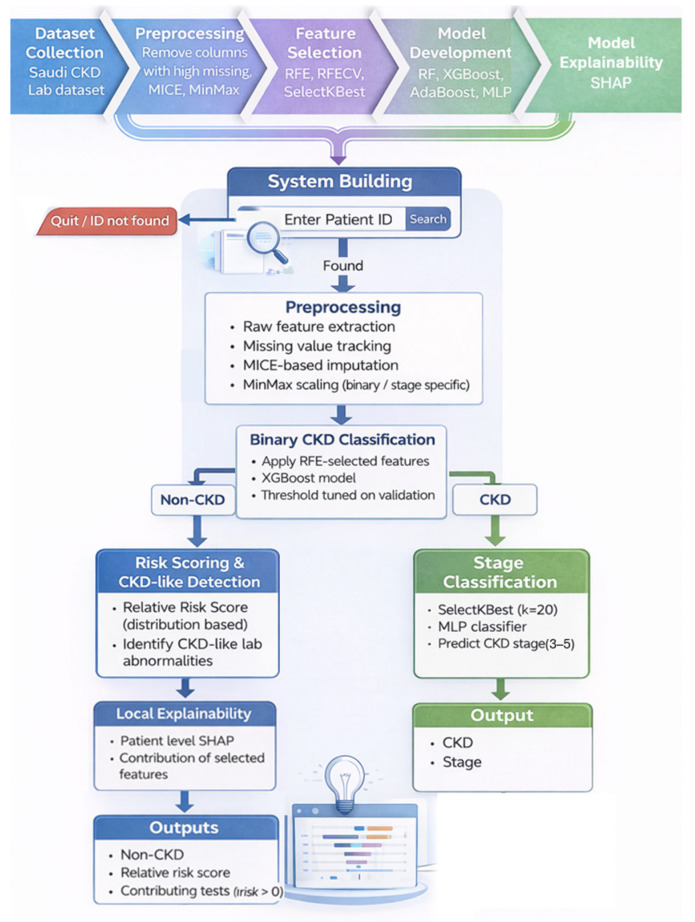
Proposed system workflow.

Based on the research limitations and future directions identified in prior studies, this work aims to address several key gaps in existing approaches to chronic kidney disease (CKD) prediction. While most previous studies focus primarily on binary disease detection or early diagnosis, the proposed approach adopts a broader and more clinically grounded strategy by jointly integrating binary, multi-stage classification, and risk-oriented assessment within a unified framework. This design enables a more realistic modeling of CKD progression while supporting early identification of individuals at elevated risk before overt disease onset. To enhance transparency and clinical trust, model explainability was incorporated as a core component of the methodology. The overall framework follows a hierarchical structure that mirrors real-world clinical decision-making: CKD presence is assessed first, disease staging is performed only for confirmed cases, and risk assessment is applied to individuals without confirmed CKD. A unified preprocessing pipeline, robust feature selection strategies, and well-validated machine learning models were employed to ensure consistency, reliability, and generalizability. Figure 1 presents an overview of the proposed system workflow.

### 3.2. Dataset

In this study, a Saudi dataset was collected from the kidney clinics at the Prince Abdul Majeed Dialysis Center in Jeddah following a systematic data collection process. A list of patient identification numbers was obtained from clinic records, and each ID was reviewed to ensure the availability of laboratory tests within the past four years, thereby guaranteeing the inclusion of recent and clinically relevant information. Patients with eligible records were included in the CKD laboratory dataset, and for each individual, the most recent laboratory test was selected. Each sample (row) in the dataset, therefore, corresponds to a unique patient, and no patient has multiple records in the dataset. Each patient was assigned a unique identifier (ID) used solely for data organization and splitting purposes. All identifiers were fully anonymized and do not contain any personally identifiable information. This strategy ensured that the dataset remained both current and representative, providing a robust foundation for predictive modeling.

**Figure 2:**
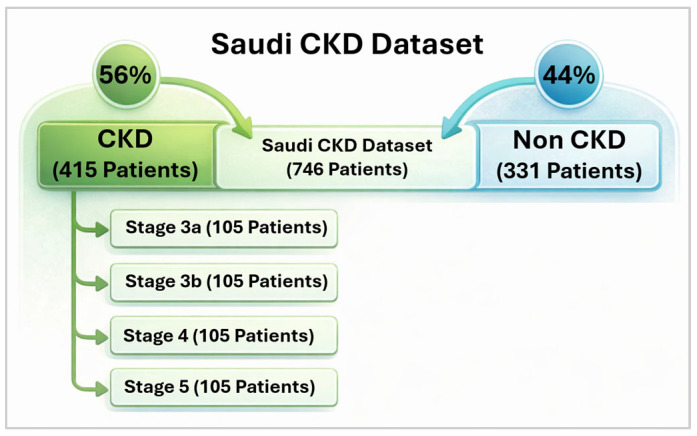
Distribution of Saudi CKD dataset.

The dataset comprises laboratory test results and demographic information for 746 participants, as illustrated in Figure 2, with a total of 60 features collected between 2021 and 2024. This dataset exceeds commonly used open-source CKD datasets in both sample size and feature dimensionality. As shown in Figure 2, CKD was defined as stages 3–5, where stage 3 was further subdivided into stages 3a and 3b, resulting in four target classes for stage classification. This definition was adopted because early stages (1–2) typically require evidence of structural abnormalities or albuminuria, which was not consistently available in routine laboratory testing. Table 1 summarizes the full dataset description and feature composition.

### 3.3. Preprocessing

#### 3.3.1. Handling Features with High Missing Values

In the preprocessing stage, the features were divided into two categories: those with more than 70% missing data and those with less than 70% missing data. The 21 features exceeding 70% of missing data were removed, leaving a total of 39 features. The removed features included: General blood pressure records, DBIL, LDH, NON-HDL, Glycated Hemoglobin, Troponin I, Iron, GGT, Lipase, Amylase, CK, CK-MB, Vitamin B12, Ferritin, Vitamin D, PTH, TSH, FT4, FT3, Specific Gravity, and Urine Glucose. These features were removed for two reasons. First, they were the least frequently recorded laboratory tests in this clinical setting, reflecting the routine testing practices at the Prince Abdul Majeed Dialysis Center, where such specialized biomarkers are not consistently ordered for all patients. Second, model evaluation showed that including these highly sparse features reduced predictive performance, while removing them led to more stable and accurate results. Therefore, excluding these features enhanced both the quality of the dataset and the reliability of the predictive modeling.

#### 3.3.2. Handling Missing Values

Remaining missing values were imputed using Multiple Imputation by Chained Equations (MICE), implemented via the IterativeImputer function in scikit-learn (version 1.6.1). This approach preserves multivariate relationships between features and is more robust than simple imputation strategies. To prevent data leakage, the imputer was fitted exclusively on the training set and subsequently applied unchanged to the validation, test, and inference datasets. The same trained imputer is reused during real-time deployment to ensure consistency and reproducibility [26]. A summary of per-feature missing rates was computed using the original dataset prior to any preprocessing steps and is provided in Supplementary Table S1.

#### 3.3.3. Data Scaling

Data scaling was performed using the MinMaxScaler, function implemented in scikit-learn (version 1.6.1), normalizing all numerical variables to the [0, 1] range. This step ensures consistent feature scales and improves the performance of models that are sensitive to feature magnitude, such as SVMs and neural networks [27]. The scaler was fitted only on the training data to prevent information leakage and was subsequently applied unchanged to the validation, test, and inference datasets.

#### 3.3.4. Data Splitting

To ensure a robust and reliable evaluation of model performance, two complementary data splitting strategies were adopted: a stratified hold-out split for system development and a 5-fold stratified cross-validation procedure for performance validation.

In both strategies, all data splits were inherently performed at the patient level, with patient IDs preserved to ensure consistent alignment across binary and stage classification tasks. This ensures that no patient appears in more than one split, completely preventing any form of data leakage between training, validation, and test sets.

For the hold-out strategy, three stratified split ratios (60/20/20, 70/15/15, and 80/10/10) were evaluated, with the 70/15/15 configuration providing the best balance between performance stability and sample adequacy. This split was used during model development and system construction.

To obtain a more robust and less split-dependent performance estimate, a 5-fold stratified cross-validation strategy was subsequently employed. Within each fold, all preprocessing steps, including MICE-based imputation, feature scaling, and feature selection, were fitted exclusively on the training portion of the data and applied unchanged to validation and test subsets within that fold. In addition, an inner validation split was derived from the training partition of each fold to tune the decision threshold for the binary classification head. The outer test fold remained completely unseen during preprocessing, feature selection, threshold tuning, and model training, ensuring unbiased evaluation.

It is important to note that cross-validation was used exclusively for performance evaluation rather than system construction. While cross-validation provides a more reliable estimate of generalization by averaging performance across multiple data partitions, real-world deployment requires a single, reproducible model pipeline. Therefore, after validating the robustness of the proposed framework through cross-validation, the final CKD risk system was constructed as a unified model using the full training configuration, ensuring consistency, reproducibility, and practical applicability in clinical settings.

### 3.4. Feature Selection

Feature selection plays an important role in machine learning by reducing computational complexity, improving predictive performance, and enhancing model interpretability [28]. In this study, several feature selection strategies were evaluated, including Recursive Feature Elimination (RFE), RFE with Cross-Validation (RFECV), Lasso regression, and correlation-based filtering, all implemented using scikit-learn (version 1.6.1). Among these, RFE and RFECV consistently achieved superior validation performance across multiple classifiers and were therefore adopted for the tree-based models. For the MLP classifier, SelectKBest was employed due to its computational efficiency and compatibility with neural network training.

For RFE-based experiments, candidate feature subset sizes (K ∈ {10, 12, 15, 18, 20, 22, 24, 26, 28, 30}) were evaluated. Feature selection was performed exclusively on the training set, and the optimal subset was selected based on validation performance using clinically relevant metrics, including F1-score and AUC. RFECV automatically determined the optimal number of features through stratified cross-validation, with a minimum constraint of five features to preserve clinically meaningful information.

For the MLP model, SelectKBest, implemented in scikit-learn (version 1.6.1), was adopted as a statistically grounded alternative, ranking features based on univariate scoring functions and selecting the top-performing subset. This strategy ensured an effective balance between dimensionality reduction, computational efficiency, and predictive performance.

### 3.5. Model Development

In this stage of the study, a unified hierarchical modeling pipeline was developed to enable fair and consistent comparison across all evaluated models. Using the same 70/15/15 master data split and identical preprocessing steps, four classifiers were examined within the two-level framework: Random Forest, XGBoost, AdaBoost, and a Multilayer Perceptron (MLP).

Recent advances in tabular deep-learning models (e.g., TabNet and transformer-based architectures) have shown promising results in structured data tasks. However, such models typically require larger datasets, increased computational resources, and more complex tuning procedures. In contrast, tree-based ensemble models such as XGBoost and Random Forest provide strong performance while maintaining a level of interpretability that is essential in medical decision-making. Therefore, the models used in this study were selected to balance predictive performance with interpretability and practical deployability in clinical settings.

Within this framework, the binary head (CKD vs. non-CKD) and the stage head (stages 3–5) were trained as separate models but evaluated on aligned patient partitions derived from the same master split. For the binary classification task, the decision threshold was optimized on the validation set to maximize balanced accuracy, ensuring an appropriate trade-off between sensitivity and specificity in the presence of class imbalance. Model performance was reported on the training, validation, and test sets using accuracy, precision, recall, F1-score, and AUC, with macro-average metrics used for the multi-class staging task.

#### 3.5.1. Training Strategy

All models were trained using a unified experimental pipeline to ensure reproducibility and prevent data leakage. A common master split and identical preprocessing steps were applied across all experiments. Feature selection was embedded within training and performed exclusively on the training data, while validation and test sets were used only for performance evaluation. Binary and stage classification models were trained independently but evaluated on aligned patient partitions, enabling fair comparison across classifiers while maintaining strict separation between training, validation, and testing phases.

#### 3.5.2. Implementation Details and Hyperparameters

The proposed hierarchical framework was implemented using the Python programming language. All experiments were conducted in a cloud-based environment using Google Colab Pro (Google LLC, Mountain View, CA, USA).

Machine learning models were developed using Python (version 3.12, Python Software Foundation, Wilmington, DE, USA) with scikit-learn (version 1.6.1), XGBoost (version 3.2.0, Distributed Computing Group, University of Washington, Seattle, WA, USA), and SHAP (version 0.51.0). Additional data processing was performed using NumPy (version 2.0.2) and Pandas (version 2.2.2).

The key hyperparameters related to model design—including feature selection size, neural network architecture, and decision threshold optimization—are summarized in Table 2. To maintain clarity and interpretability, only the most relevant non-default hyperparameters are reported, while all other parameters were retained at their default settings.

#### 3.5.3. Hierarchical Classification

The hierarchical classification framework decomposes CKD classification into two sequential stages: binary classification (CKD vs. non-CKD), followed by disease staging only for confirmed CKD cases. This design mirrors real-world clinical workflows, where disease presence is first established before severity assessment [29]. By restricting stage classification to CKD-positive individuals, the framework reduces unnecessary predictions, limits error propagation, and improves clinical coherence. This hierarchical structure enhances interpretability and ensures that each model focuses on a clearly defined and clinically meaningful task [30].

#### 3.5.4. Threshold Tuning for Binary Classification

Instead of relying on a fixed decision threshold (e.g., 0.5) for binary CKD classification, the decision threshold was optimized using the validation set. Predicted probabilities were evaluated across multiple candidate thresholds, and the threshold maximizing balanced accuracy was selected to account for class imbalance. The optimized threshold was then applied unchanged to the test set to avoid optimistic bias and improve generalization. Within the proposed hierarchical framework, this threshold optimization ensures clinically appropriate sensitivity at the initial CKD classification stage before proceeding to disease staging.

#### 3.5.5. Model Explainability

To enhance transparency and support clinical interpretability, model explainability was explicitly integrated into the experimental workflow using SHapley Additive exPlanations (SHAP) [31]. SHAP enables decomposition of model predictions by quantifying the contribution of each laboratory feature toward higher or lower CKD risk, thereby mitigating black-box behavior and improving model interpretability [30]. Given that the primary classifiers in this study are tree-based, SHAP’s TreeExplainer, as implemented in the SHAP library (version 0.51.0), was employed due to its computational efficiency and theoretically grounded feature attributions. To balance interpretability with runtime efficiency, SHAP values were computed using a controlled subset of the training data.

**Figure 3:**
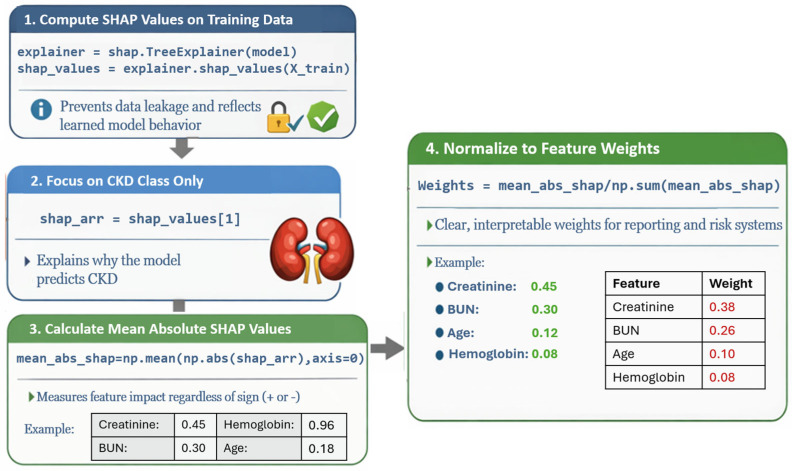
SHAP analysis pipeline.

For the binary CKD classification task, SHAP values were calculated with a specific focus on the positive (CKD) class to characterize features driving disease-related predictions. Global feature importance was quantified by computing the mean absolute SHAP value for each feature and normalizing these values into relative contribution weights. This normalization enabled direct comparison across features and facilitated downstream integration with the proposed risk assessment system. The complete SHAP pipeline is illustrated in Figure 3.

## 4. Hierarchical Classifiers Results

Model performance was further validated using 5-fold stratified cross-validation to ensure robustness and reduce dependence on a single data split. Table 3 presents the performance of the proposed hierarchical framework evaluated using 5-fold stratified cross-validation. The reported values represent the mean and standard deviation across folds.

The 5-fold cross-validation results demonstrate that the proposed hierarchical framework achieves strong and stable performance across different data partitions. For the binary classification task, the model consistently achieved high discriminative ability, with a mean AUC of 0.991 and a balanced accuracy of 0.964, indicating reliable separation between CKD and non-CKD cases. For stage classification, the model achieved a mean accuracy of 0.798 and a macro F1-score of 0.798, reflecting robust performance in multi-class CKD staging. Although the stage classification task is inherently more challenging, the results remain consistent across folds, as indicated by relatively low standard deviation values. Macro-averaged metrics were used for the stage classification task to ensure that all classes were equally represented in the evaluation. Unlike micro-averaging, which may be dominated by majority classes, macro-averaging assigns equal importance to each class, thereby providing a more clinically meaningful and fair assessment of model performance across all CKD stages. Overall, the cross-validation results confirm that the proposed framework is not dependent on a specific data split and maintains stable performance across different subsets of the dataset, supporting its robustness and generalizability. In addition, the consistency of selected features across folds further supports the stability of the learned clinical patterns.

The cross-validation results are generally consistent with those reported in Table 4, which were obtained using the 70/15/15 hold-out data split, particularly for the binary classification task, where only minor differences were observed. For stage classification, a moderate decrease in performance was observed under cross-validation compared to the 70/15/15 split. This difference is expected due to the increased evaluation variability introduced by multiple folds and the more stringent evaluation setting.

Table 4 summarizes the comparative predictive performance of the evaluated classifiers under the proposed hierarchical framework, considering different feature selection strategies for both the binary head (CKD vs. non-CKD) and the stage head (CKD staging). In parallel, Table 5 reports the corresponding subsets of selected features obtained using each feature selection method across the evaluated models. Together, these tables provide a comprehensive assessment of both predictive accuracy and model interpretability within the proposed framework. The reported runtime in Table 4 reflects the total end-to-end execution time, including preprocessing, feature selection, model training, and evaluation. This provides a more realistic estimate of the computational cost of the full pipeline.

As shown in Table 4, all ensemble-based models achieved consistently high performance in the binary classification task, with accuracy values ranging between 0.96 and 0.97 and macro-AUC values approaching 0.99 across most experimental configurations. Random Forest, XGBoost, and MLP demonstrated strong discriminative capability, indicating that routine laboratory features contain sufficient predictive information to reliably distinguish CKD from non-CKD patients. Notably, binary classification performance remained stable across different feature selection strategies, reflecting the robustness of the hierarchical framework in capturing disease-related patterns.

In contrast, the stage classification head posed a more challenging task, as reflected by comparatively lower performance across all evaluated models. This reduction is consistent with the increased class granularity and overlap between adjacent CKD stages. Among the evaluated configurations, AdaBoost combined with RFECV and MLP combined with SelectKBest achieved the strongest staging performance, with accuracies reaching approximately 0.85 and macro-AUC values in the range of 0.96–0.97. Random Forest-based staging models exhibited comparatively lower accuracy (≈0.69–0.78) under aggressive feature elimination, indicating greater sensitivity to dimensionality reduction in multi-class staging tasks.

Feature selection improved computational efficiency in several configurations without substantially compromising predictive performance; however, this effect was not uniform across all models and tasks. For example, for the MLP classifier, RFE reduced runtime from 44 s to 8 s while increasing accuracy from 94% to 97%. In contrast, certain stage-classification models exhibited slight performance reductions under aggressive feature elimination. These findings indicate that the impact of feature selection is model- and task-dependent, supporting the need for systematic evaluation prior to deployment in real-world clinical environments.

### External Validation on a Public Dataset

To evaluate the generalizability of the proposed framework beyond the region-specific dataset, an additional experiment was conducted using a publicly available dataset (UCI CKD dataset) [19]. The dataset consists of 400 patient records (150 non-CKD and 250 CKD) with 24 clinical features and binary labels (CKD vs. non-CKD) and is widely used as a benchmark for CKD prediction tasks.

Due to the absence of reliable stage annotations and cohort-specific risk structure in public datasets, the evaluation was limited to the binary classification component of the proposed hierarchical framework, which represents the primary entry point of the system.

The same preprocessing pipeline, including MICE-based imputation and MinMax scaling, was applied, along with the same feature selection and modeling strategy (XGBoost with RFE). In addition, a feature selection sensitivity analysis was conducted using a grid search over the number of selected features (k = 5 to 18) to identify the optimal feature subset size.

Table 6 presents the external validation results on the UCI CKD dataset for the binary classification component of the proposed framework, including both hold-out and 5-fold cross-validation performance using the optimal number of selected features (k = 8).

The results demonstrated strong performance, achieving an accuracy of 0.983 and an AUC of 1.000 on the hold-out test set. Furthermore, 5-fold cross-validation confirmed stable performance, with a mean accuracy of 0.987 ± 0.009 and AUC close to 1.000 across folds.

A sensitivity analysis showed that model performance remained highly stable across a wide range of feature counts, with minimal variation in F1-score. The best performance was achieved at k = 8; however, similar performance was observed for larger feature sets, indicating that the model is not highly sensitive to the exact number of selected features. Therefore, k = 8 was selected as it provides a good balance between model simplicity and predictive performance.

This suggests that the selected features effectively capture the core discriminative signal, even with a reduced feature set. The high performance observed on the UCI dataset is consistent with prior studies and reflects the relatively well-separated nature of the dataset.

Overall, these findings indicate that the binary detection component of the proposed framework generalizes well to external datasets and is not limited to region-specific characteristics.

## 5. Discussion of Hierarchical Classifiers Results

The experimental results demonstrate the effectiveness of the proposed hierarchical framework in capturing clinically meaningful patterns from routinely available laboratory data. Overall, the evaluated ensemble-based models achieved consistently strong performance in the binary classification task, with accuracy values ranging between 0.96 and 0.97 and macro-AUC values approaching 0.99 across most experimental configurations. This indicates that routine laboratory profiles contain sufficient discriminative information to reliably distinguish CKD from non-CKD patients. Such findings are clinically intuitive, as moderate-to-advanced CKD stages are typically associated with pronounced biochemical abnormalities, including elevated serum creatinine, abnormal urea levels, hematologic disturbances, and proteinuria, which are well reflected in standard laboratory tests. The observed robustness across different feature selection strategies further supports the stability and generalizability of the proposed framework, reinforcing its suitability for real-world clinical screening.

In contrast, the stage classification task proved to be more challenging, as reflected by comparatively lower predictive performance across all models. This behavior is expected given the increased class granularity and the inherent overlap in laboratory profiles between adjacent CKD stages, particularly between stages 3a and 3b. Among the evaluated configurations, AdaBoost combined with RFECV and MLP combined with SelectKBest achieved the strongest staging performance, with accuracies reaching approximately 0.85 and macro-AUC values in the range of 0.96–0.97. These results suggest that both ensemble-based learners and neural networks are capable of modeling the complex, nonlinear relationships required for fine-grained disease stratification when supported by effective feature selection. In contrast, Random Forest-based staging models exhibited comparatively lower accuracy under aggressive feature elimination, indicating higher sensitivity to dimensionality reduction in multi-class scenarios. Collectively, these findings highlight that CKD stage differentiation requires more nuanced modeling strategies than binary classification and benefits substantially from careful feature selection and algorithm pairing.

**Figure 4:**
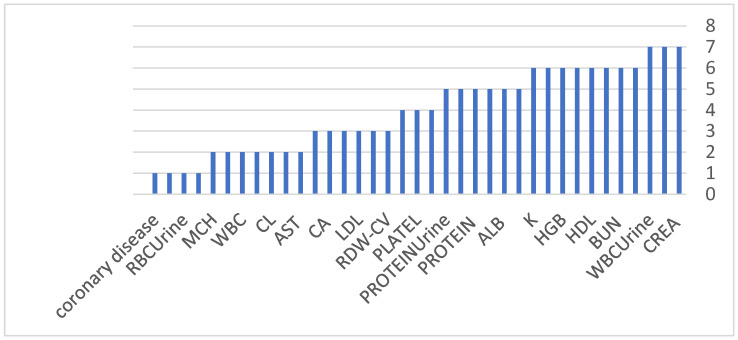
Selected feature frequency in binary head.

**Figure 5:**
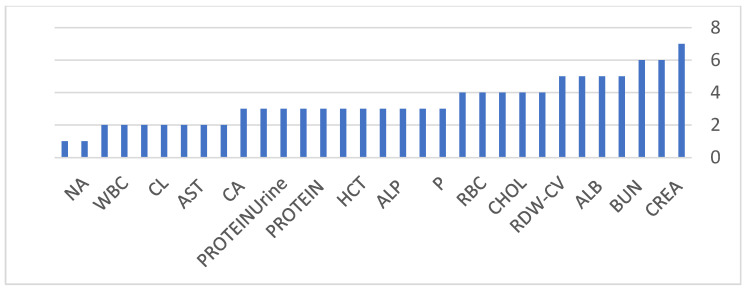
Selected feature frequency in stage head.

An important observation across all experiments is the consistency of selected features across models and feature selection strategies. Core clinical markers, including serum creatinine (CREA), blood urea nitrogen (BUN), hemoglobin (HGB), hematocrit (HCT), red blood cell indices, and urine protein, were repeatedly identified as dominant predictors for both binary classification and staging tasks. This consistency reinforces the clinical validity of the proposed framework and its alignment with established nephrology knowledge, as these biomarkers are widely recognized indicators of impaired renal function, anemia of chronic disease, and renal-associated metabolic dysregulation. As illustrated in Figure 4 and Figure 5, the frequency of feature selection for the binary and stage heads further confirms the stability of clinically meaningful predictors across different modeling strategies, strengthening confidence in the biological plausibility of the learned patterns.

Beyond predictive accuracy, feature selection contributed to reduced computational runtime and improved model efficiency in several configurations without compromising performance. This aspect is particularly important for real-world deployment, where rapid inference and low computational overhead are essential for seamless integration into clinical workflows. Compared with prior studies that often rely on extensive feature sets or specialized biomarkers, the proposed framework demonstrates that robust and interpretable CKD prediction can be achieved using a compact subset of routinely collected laboratory variables, enhancing feasibility in resource-constrained clinical environments.

When contextualized within the existing literature, the present findings extend prior work in several important directions. While many earlier studies focus exclusively on binary CKD detection or early prediction using longitudinal data, the proposed hierarchical framework jointly addresses disease presence, severity stratification, and individualized risk assessment. This integrated design more closely reflects real-world clinical reasoning, where disease confirmation precedes staging and targeted monitoring. Furthermore, the explicit incorporation of explainability through SHAP strengthens clinical trust by enabling transparent interpretation of model predictions, addressing a key limitation of many black-box AI systems reported in earlier research.

## 6. Proposed CKD Risk Assessment System

### 6.1. System Overview

This section presents the proposed CKD risk assessment system from a deployment and clinical workflow perspective. The system operates on routinely available laboratory data to support CKD screening and decision-making, performing disease classification first and stage classification (stages 3–5) only when CKD is identified. In addition to classification, a dedicated risk stratification component is applied to individuals predicted as non-CKD, with the goal of identifying CKD-like laboratory patterns and highlighting clinically relevant signals for further review. The overall system design prioritizes three key objectives: (1) robustness to real-world data missingness and variability, (2) clinically coherent hierarchical decision-making aligned with standard diagnostic workflows, and (3) controlled interpretability and decision support that highlights CKD-like signals while minimizing unnecessary clinical burden.

### 6.2. Input Handling and System Workflow

Each patient record is uniquely indexed using a patient identifier (ID), which serves as the entry point to the proposed system. Upon receiving an input request, the system first verifies the existence and structural validity of the provided ID. If the identifier is not found in the database or if the incoming payload lacks the required format, the request is rejected, and an informative validation message is returned.

For valid patient records, the corresponding laboratory data are passed through a unified inference preprocessing module, which applies the same imputation and scaling procedures used during model training. The processed record is then forwarded to the binary classification head, where a tuned decision threshold is applied to determine whether the patient is likely to have CKD.

If the predicted probability exceeds the decision threshold, the patient is classified as CKD-positive and the record proceeds to the stage classification head, which assigns a disease stage ranging from stage 3 to stage 5. Conversely, if the patient is classified as non-CKD, the system does not perform stage classification. Instead, a dedicated risk assessment module is activated, computing a normalized risk score and conditionally highlighting contributing laboratory features based on predefined clinical rules.

**Figure 6:**
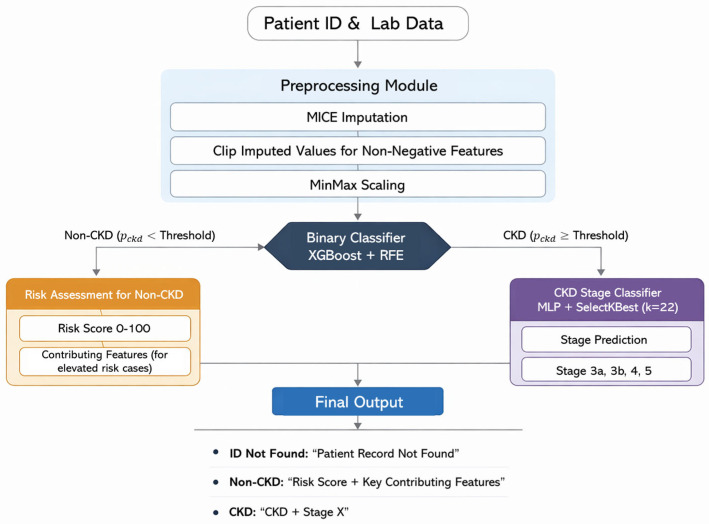
Risk system framework.

The final system output is generated according to the decision path taken, providing one of three outputs: (i) a CKD diagnosis with stage assignment, (ii) a non-CKD risk assessment with interpretable laboratory indicators, or (iii) an informative validation message when applicable. Figure 6 illustrates the complete architecture of the proposed system, detailing the sequential flow from input validation and preprocessing to hierarchical classification and final output generation.

### 6.3. Unified Inference Preprocessing (Deployment Readiness)

To ensure robustness and clinical reliability under real-world deployment conditions, a unified inference preprocessing pipeline was designed to mirror training-time transformations while explicitly addressing practical constraints encountered in routine laboratory data. For each incoming patient record, a raw laboratory feature vector is first extracted. Missing values are tracked internally to distinguish between originally observed and imputed measurements, enabling clinically transparent downstream interpretation.

Missing data is handled using MICE imputation. To maintain physiological plausibility, a conservative non-negativity constraint is applied to clinically non-negative laboratory features. Features exhibiting negligible proportions of negative values in the training data are treated as physiologically non-negative. After imputation, this constraint is enforced only on imputed entries, while all originally observed measurements are preserved unchanged. This approach prevents the introduction of implausible values without distorting the original data distribution or over-constraining the imputation process.

Finally, to accommodate task-specific distributions within the hierarchical framework, two independent MinMax scalers are employed: one fitted on training data for the binary CKD classification head, and another fitted on training data restricted to CKD cases for the staging head. This separation avoids unintended coupling between tasks and ensures stable and consistent inference behavior across both classification stages.

### 6.4. Hierarchical Classification Framework

This section describes the inference-time operation of the hierarchical classification framework and explains how model-level design choices were guided by deployment-oriented considerations rather than training performance alone.

#### 6.4.1. Binary Head Model Selection

Several candidate models demonstrated similarly high predictive performance for binary CKD detection. Therefore, final model selection was guided by two deployment-relevant criteria: computational efficiency and the behavior of the risk-based identification mechanism for non-CKD individuals. Flagged non-CKD cases refer to individuals assigned a non-zero CKD risk score under the proposed risk assessment mechanism. This criterion reflects how each model identifies borderline cases that fall below the decision threshold yet receive non-zero CKD risk scores. Such cases serve as an early warning layer; however, excessive identification may increase false alerts, leading to unnecessary clinical burden, whereas overly restrictive behavior may suppress subtle risk signals.

Based on the comparative analysis (Table 7), the MLP-based model was excluded due to substantially longer runtime, limiting its suitability for real-time integration. The Random Forest model, although performant, produced a markedly higher number of flagged non-CKD cases, suggesting heightened sensitivity that could increase unnecessary clinical alerts and downstream testing. Among the remaining candidates, XGBoost-based models demonstrated comparable behavior. XGBoost with RFE was selected as the final binary head due to its favorable runtime, stable risk behavior, and simpler feature selection procedure. Notably, alternative configurations such as Random Forest remain viable for future extensions where higher sensitivity may be clinically desirable.

#### 6.4.2. Binary Head Architecture and Decision Logic

The selected binary head outputs a continuous probability representing CKD likelihood. At inference time, the validation-optimized decision threshold is used as a hierarchical decision gate that governs downstream system behavior. Patients exceeding this threshold are classified as CKD and forwarded to the stage classification module, whereas patients below the threshold are classified as non-CKD and redirected to the risk assessment pathway. This threshold-based gating mechanism ensures clinically appropriate sensitivity at the first decision point of the system and enables a coherent transition between detection, staging, and risk-oriented analysis.

#### 6.4.3. Stage Head Architecture

After applying task-specific feature scaling, a univariate feature selection step is performed using a SelectKBest strategy to retain the 22 most informative laboratory measurements for CKD stage differentiation. At the second level of the hierarchy, stage prediction is formulated as a multiclass classification task restricted to CKD-positive cases. The stage head operates as a multiclass classifier over the target CKD stages and produces a probability distribution across all candidate stages for each patient. The final predicted stage is then assigned using a maximum-probability decision rule, i.e., the stage with the highest predicted probability.

### 6.5. Risk Score Computation for Non-CKD Predictions

For patients predicted as non-CKD (y=0), no CKD stage is assigned. However, a non-CKD classification does not necessarily imply absence of risk, particularly for borderline cases whose laboratory profiles partially resemble CKD-associated patterns.

The binary model outputs a continuous probability $p_{\mathrm{CKD}}\in \left[0,1\right]$ reflecting the likelihood of CKD. While a tuned decision threshold determines whether CKD is formally declared for staging, the proposed system extends beyond a strict threshold-based interpretation by introducing a continuous risk score that contextualizes $p_{\mathrm{CKD}}$ relative to probability distributions learned from the training population.

Let $P_{0}$ denote the set of predicted CKD probabilities for training non-CKD samples, and $P_{1}$ denote the corresponding set for training CKD samples. The 95th percentile of $P_{0}$, denoted as $Q_{0.95}\left(P_{0}\right)$, represents the upper tail of non-CKD probabilities, capturing high-normal cases that lie close to the decision boundary. Conversely, the 5th percentile of $P_{1}$, denoted as $Q_{0.05}\left(P_{1}\right)$, represents the lower tail of CKD probabilities, capturing low-confidence CKD cases. Together, these anchors define a clinically meaningful transition region between non-CKD and CKD distributions. Using quantiles rather than extreme values helps reduce the influence of outliers and prevents overly sensitive risk inflation.

**Figure 7:**
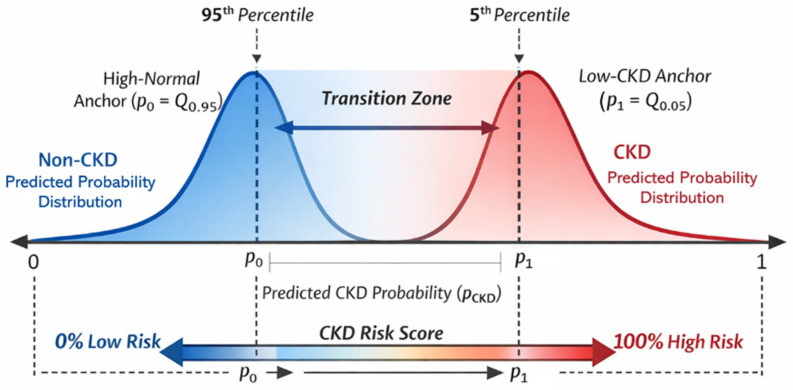
Quantile-based risk score mapping between non-CKD and CKD probability distributions.

The patient’s predicted probability is mapped onto a normalized risk scale ranging from 0 to 100, reflecting proximity to the CKD decision boundary within the learned model space. Specifically, if $p_{\mathrm{CKD}}\leq p_{0}$, the patient lies within the typical non-CKD range, and the assigned risk score is 0. If $p_{\mathrm{CKD}}\geq p_{1}$, the patient approaches the lower boundary of the CKD probability distribution, and the assigned risk score approaches 100. Intermediate values represent a gradual increase in similarity to CKD-like patterns, providing an early-warning signal without triggering a formal CKD-positive classification or unnecessary disease staging, thereby preserving diagnostic specificity and reducing unwarranted clinical interventions. Figure 7 illustrates the risk score computation, highlighting the transition zone between non-CKD and CKD probability distributions and the resulting continuous risk mapping. Density curves are shown for illustrative purposes and do not assume a specific parametric distribution.

From a clinical perspective, the continuous risk score is not intended to function as a rigid diagnostic cutoff but rather as a decision-support indicator. For example, patients with low risk scores (e.g., <20) may be considered within the typical non-CKD range and require no additional evaluation beyond routine care. In contrast, patients with moderate risk scores (e.g., 20–50) may warrant closer monitoring or repeat laboratory testing, while higher risk scores (e.g., >50) may prompt targeted investigation, such as urine albumin-to-creatinine ratio (UACR) testing or referral for further nephrology assessment.

It is important to note that these thresholds are provided as illustrative examples rather than fixed clinical rules. Because the risk score is derived from cohort-specific probability distributions, the optimal decision thresholds may vary depending on the target population, clinical setting, and screening objectives.

From a sensitivity perspective, lower thresholds increase sensitivity for early risk detection but may lead to higher rates of false alerts, whereas higher thresholds improve specificity at the expense of potentially missing borderline cases. This trade-off allows the proposed system to be flexibly adapted to different clinical priorities, such as population-level screening versus specialist referral settings.

### 6.6. CKD-like Pattern Detection

While the risk score quantifies a patient’s proximity to CKD at the population level, the CKD-like pattern detection module translates this probabilistic risk into feature-level signals by identifying laboratory measurements that resemble CKD-specific distributions. These CKD-like indicators are subsequently integrated with SHAP-based contributions to generate patient-level explanations, as described in Section 6.7.

#### 6.6.1. Data-Driven Reference Ranges

For interpretability, the system computes reference ranges for each laboratory feature using non-CKD patients from the training set. These ranges represent the central distribution of non-CKD values within the study cohort and are not intended to replace standard clinical laboratory reference intervals. Instead, they provide a cohort-specific baseline that reflects the expected laboratory behavior of individuals without CKD in the studied population. Importantly, the system does not rely on conventional clinical cutoffs to determine abnormality. Rather than assessing whether a laboratory value is elevated or reduced relative to standard medical reference ranges, the system focuses on whether the value has shifted toward patterns that are characteristic of CKD patients.

#### 6.6.2. Cutpoint-Based Abnormality Detection

To determine whether a laboratory value exhibits CKD-like behavior, the system compares feature distributions between non-CKD and CKD samples within the training data. For each feature, the direction of change associated with CKD progression is first identified. A data-driven cutpoint is then defined such that values beyond this threshold reflect increased similarity to the CKD population. A laboratory test is flagged as CKD-like only when the patient’s value crosses this cutpoint in the disease-associated direction.

This distinction is clinically critical. A laboratory value may fall outside standard clinical reference ranges yet still remain within the typical distribution of non-CKD individuals and therefore would not be considered CKD-like by the system. Conversely, a value that is still clinically “normal” may be flagged if it closely resembles the distribution observed among CKD patients. By emphasizing similarity to CKD-specific patterns rather than absolute clinical abnormality, this mechanism enables early identification of subtle disease-related signals and translates probabilistic model outputs into clinically meaningful and interpretable alerts.

Table 8 presents representative examples of the derived data-driven CKD-like cutpoints, while the complete set of thresholds for all laboratory features is reported in Appendix A.

### 6.7. Explainability Framework

#### 6.7.1. Global Explainability (Model-Level)

At the global level, SHAP is used to quantify how much each laboratory feature contributes—on average—to the binary model’s predicted CKD probability across the training population. For global interpretation, the mean absolute SHAP value is computed for each feature across many samples. This reflects the typical magnitude of that feature’s influence on the model output, regardless of whether it increases or decreases risk in individual cases. These magnitudes are then normalized into weight_norm so that features can be compared on a common scale and ranked.

**Figure 8:**
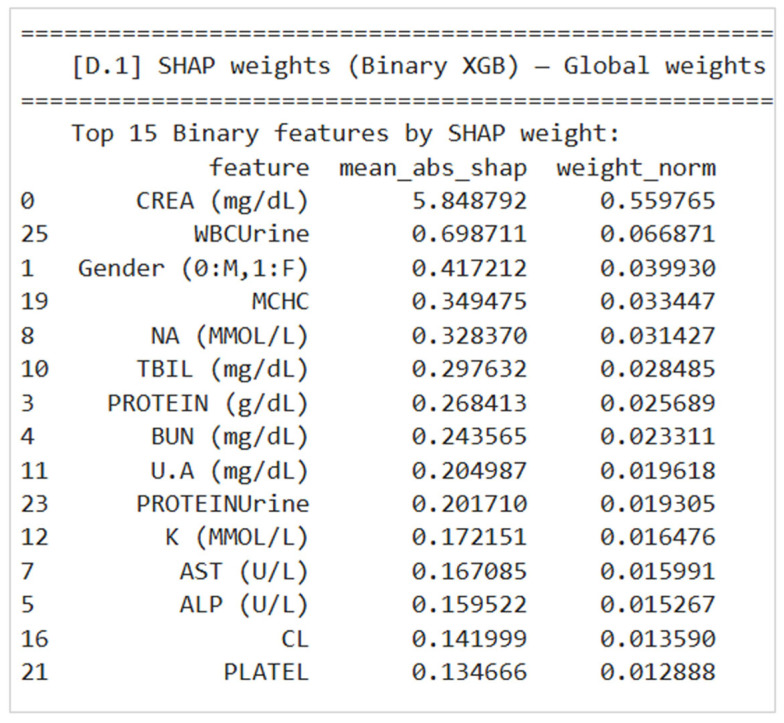
Global SHAP weights.

This is reflected directly in the system output: the section “Top 15 Binary features by SHAP weight” lists each feature with its mean_abs_shap and weight_norm, showing which measurements consistently drive model decisions. For example, CREA (mg/dL) has the largest normalized weight, indicating it is the most influential feature overall for distinguishing CKD from non-CKD, followed by urine and hematologic markers such as WBCUrine and PROTEINUrine. Figure 8 presents the Top 15 Binary features by SHAP weight, matching the printed output table.

#### 6.7.2. Local Patient-Level Explainability (Case-Level)

**Figure 9:**
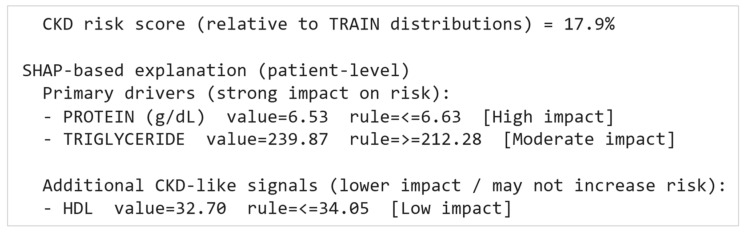
Local SHAP interpretation of patient-level feature contributions.

While global SHAP explains what the model generally relies on, local SHAP explains why the model produced a specific probability for an individual patient. For a given patient, SHAP produces a signed contribution for every selected feature: a positive SHAP contribution pushes the prediction toward CKD (increases p(CKD)), while a negative SHAP contribution pushes the prediction away from CKD (decreases p(CKD)). Thus, for each patient, the model output can be expressed as the baseline probability plus the sum of feature-level SHAP contributions. To make the explanation clinically actionable, the system converts raw SHAP contributions into an interpretable summary aligned with the printed output in Figure 9.

Risk score output (probability-based):

The system first reports p(CKD) and the binary decision (CKD vs non-CKD). For non-CKD cases, it additionally reports a risk score (0–100) anchored to training distributions, expressing how close the patient’s probability is to typical CKD patterns (e.g., “CKD risk score = 17.9%”).

Primary drivers (SHAP + CKD-like pattern alignment):

Among all features, the system prioritizes those that (i) have positive SHAP contributions (i.e., they increase risk) and (ii) are CKD-like according to the cohort-derived cutpoint rule (i.e., the patient value crosses a CKD-pattern threshold). These are prioritized because they represent features that both increased the model’s predicted risk and resemble CKD distributions in the cohort. In the example output, this yielded:

PROTEIN (g/dL) with its value and cutpoint rule, flagged as High impact
TRIGLYCERIDE with its value and cutpoint rule, flagged as Moderate impact

Here, the impact label (High/Moderate/Low) is derived from the feature’s relative SHAP weight within the patient’s explanation—specifically, how large its contribution is compared with the sum of absolute contributions for that patient.

Additional CKD-like signals:

The system also reports other CKD-like features that meet the cohort cutpoint criteria, even if they have weaker contributions or do not strongly increase risk. This is important because a feature may resemble CKD patterns but have a small net effect on the final probability due to interactions with other variables. In the example, HDL was flagged as: HDL value = 32.70, rule ≤ 34.05 [Low impact].

Overall, this approach supports the system’s goal: the explanation does not merely list clinically “abnormal” labs but highlights tests that appear CKD-like relative to the dataset’s CKD population and indicates whether they meaningfully influenced the model’s output.

#### 6.7.3. Controlled Explanation Policy

To reduce unnecessary alarm and limit cognitive burden, explanations are selectively displayed based on the clinical pathway. If the patient is classified as CKD, the system reports the CKD decision and the predicted stage (Stages 3–5) without displaying risk-style explanations. If the patient is classified as non-CKD, the system reports (i) p(CKD), (ii) the risk score, and (iii) SHAP-based drivers only when the risk score is non-zero, ensuring that explanations appear primarily when there is a meaningful risk signal. This policy balances transparency with practicality: the clinician receives detailed explanations only when they add value for decision support and follow-up consideration.

### 6.8. Deployment and Reproducibility

To ensure reproducible inference and safe clinical deployment, the complete system pipeline is preserved as serialized artifacts. These include the trained imputers, scalers, feature selectors, classification models, optimized decision thresholds, risk score anchors, CKD-like cutpoints, and patient-level split identifiers. By freezing all preprocessing and decision components learned during validation, the system guarantees that future predictions follow identical data transformations and inference logic. This design supports reproducibility, auditability, and consistent behavior across research replication and real-world deployment scenarios.

## 7. Conclusions

In this study, a risk-oriented, explainable, and hierarchical framework for classifying chronic kidney disease (CKD) using real-world laboratory data from a Saudi population was presented. Instead of relying solely on traditional binary classification or attempting to directly predict all CKD stages, the proposed framework decomposes the classification task into clinically meaningful steps, enabling reliable detection, appropriate staging, and risk-oriented assessment.

The experimental results demonstrate that the proposed approach achieves strong and consistent performance across both CKD detection and staging tasks. In particular, the results remained stable across different evaluation settings, including both hold-out and cross-validation experiments, indicating that the framework is not dependent on a specific data split. Furthermore, the hierarchical structure contributed to reducing clinically implausible predictions by ensuring that stage classification is performed only for confirmed CKD cases.

A key strength of the proposed framework lies in its emphasis on interpretability and prevention. Through the integration of SHAP-based explainability, the system provides transparent insights into how individual laboratory measurements influence predictions. In addition, the proposed risk scoring mechanism enables the identification of individuals with elevated CKD risk using routine laboratory tests, supporting earlier referral and closer clinical monitoring.

Overall, the findings suggest that the proposed framework offers a clinically aligned and practically deployable approach for CKD detection, staging, and risk assessment. However, further validation on larger multi-center datasets is required to confirm generalizability across diverse populations and healthcare settings.

## 8. Limitations and Future Work

Despite the encouraging results, several limitations should be acknowledged. The dataset, although substantially larger and more diverse than commonly used public benchmarks, was derived from a single regional healthcare system, which may limit external generalizability. Validation across multi-center and multinational cohorts is therefore necessary to assess robustness across diverse populations and healthcare settings. Accordingly, future work will explicitly focus on multi-center external validation using independent cohorts from different hospitals and healthcare systems. In addition, the current framework relies on cross-sectional laboratory snapshots. Incorporating longitudinal laboratory measurements may enhance early risk stratification and improve the prediction of disease progression dynamics. Finally, prospective clinical evaluations will be essential to determine the real-world impact of the proposed system on diagnostic accuracy, referral timing, and patient outcomes. Future work will therefore focus on external validation, integration of longitudinal data, and implementation within clinical decision support systems to further strengthen the translational applicability of the framework in preventive nephrology and population health.

## Figures and Tables

**Table 1:** Dataset description.

Category	# of Features	Features
Basic Information	3	ID, Gender, Age
Clinical Conditions	5	Hypertension, Systolic Blood Pressure (Systolic_BP), Diastolic Blood Pressure (Diastolic_BP), Diabetes Mellitus, Coronary Artery Disease
Biochemistry Tests	31	Creatinine, Protein, Blood Urea Nitrogen (BUN), Albumin (ALB), Alkaline Phosphatase (ALP), Alanine Aminotransferase (ALT), Aspartate Aminotransferase (AST), Calcium (Ca), Cholesterol (CHOL), Glucose (Glu), Sodium (Na), Phosphorus (P), Total Bilirubin (TBIL), Direct Bilirubin (DBIL), Uric Acid (UA), Potassium (K), Magnesium (Mg), Lactate Dehydrogenase (LDH), Non-High-Density Lipoprotein Cholesterol (Non-HDL), High-Density Lipoprotein Cholesterol (HDL), Low-Density Lipoprotein Cholesterol (LDL), Glycated Hemoglobin, Chloride (Cl), Troponin I, Iron, Triglycerides, Gamma-Glutamyl Transferase (GGT), Lipase, Amylase, Creatine Kinase (CK), Creatine Kinase-MB (CK-MB)
Hematology Tests	9	Hematocrit (HCT), Hemoglobin (HGB), Mean Corpuscular Hemoglobin (MCH), Mean Corpuscular Hemoglobin Concentration (MCHC), Mean Corpuscular Volume (MCV), Platelet Count (PLT), Red Blood Cell Count (RBC), White Blood Cell Count (WBC), Red Cell Distribution Width-Coefficient of Variation (RDW-CV)
Hormone Tests	7	Vitamin B12, Ferritin, Vitamin D, Thyroid Stimulating Hormone (TSH), Free Thyroxine (FT4), Free Triiodothyronine (FT3), Parathyroid Hormone (PTH)
Urine Tests	5	Specific Gravity, Protein, Glucose, Red Blood Count (RBC), White Blood Cell Count (WBC)
Target class (classification)	1	0: Non-CKD, 1: CKD Stage 3a, 2: CKD Stage 3b, 3: CKD Stage 4, 4: CKD Stage 5

**Table 2:** Key model hyperparameters (customized settings).

Component	Configuration
Binary Model	XGBoost with RFE (26 selected features)
Decision Strategy	Threshold optimized on validation set (range: 0.10–0.90, step = 0.01, optimized using balanced accuracy)
Stage Model	MLP with SelectKBest (22 selected features)
MLP Architecture	Hidden layers (128, 64); max_iter = 700

**Table 3:** Performance of the proposed hierarchical CKD framework evaluated using 5-fold stratified cross-validation (mean ± standard deviation).

Head	Accuracy	Precision (Macro)	Recall (Macro)	F1-Score (Macro)	AUC
Binary	0.965 ± 0.018	0.965 ± 0.025	0.973 ± 0.033	0.969 ± 0.017	0.991 ± 0.010
Stage	0.798 ± 0.041	0.800 ± 0.045	0.799 ± 0.041	0.798 ± 0.043	0.948 ± 0.016

**Table 4:** Classifier results.

Feature Selection Method	Head	Accuracy	Precision (Macro)	Recall (Macro)	F1-Score (Macro)	AUC	End-to-End Runtime
Random Forest							
All features	Binary	0.96	0.96	0.96	0.96	0.99	24 s
	Stage	0.77	0.80	0.77	0.78	0.93	
RFE	Binary	0.97	0.97	0.98	0.97	0.99	54 s
	Stage	0.69	0.70	0.69	0.69	0.91	
RFECV	Binary	0.96	0.96	0.97	0.96	0.99	7 min
	Stage	0.71	0.71	0.71	0.71	0.90	
XGBoost							
All features	Binary	0.96	0.96	0.97	0.96	0.98	5 s
	Stage	0.81	0.81	0.81	0.81	0.94	
RFE	Binary	0.97	0.97	0.97	0.97	0.99	51 s
	Stage	0.79	0.80	0.79	0.79	0.93	
RFECV	Binary	0.97	0.97	0.97	0.97	0.99	37 s
	Stage	0.79	0.80	0.79	0.79	0.94	
AdaBoost							
All features	Binary	0.96	0.96	0.96	0.96	0.99	12 min
	Stage	0.84	0.85	0.84	0.84	0.97	
RFE	Binary	0.96	0.95	0.96	0.96	0.98	54 s
	Stage	0.81	0.81	0.81	0.81	0.96	
RFECV	Binary	0.96	0.96	0.96	0.96	0.99	6 min
	Stage	0.85	0.87	0.86	0.85	0.96	
MLP							
All features	Binary	0.94	0.94	0.94	0.94	0.99	44 s
	Stage	0.85	0.85	0.85	0.85	0.95	
SelectK-Best	Binary	0.97	0.97	0.97	0.97	0.98	8 s
	Stage	0.85	0.86	0.86	0.86	0.96	

**Table 5:** Selected features.

Feature Selection Method	Head	Selected Features
Random Forest		
RFE	Binary	22: CREA, Age, hypertension, BUN, ALB, ALP, CA, CHOL, P, TBIL, U.A, K, Mg, HDL, TRGLYCERIDE, HCT, HGB, RBC, RDW-CV, PROTEINUrine, RBCUrine, WBCUrine
	Stage	18: CREA, BUN, ALB, ALP, ALT, CHOL, P, HDL, LDL, CL, TRIGLYCERIDE, HCT, HGB, PLATEL, RBC, WBC, RDW-CV, PROTEINUrine
RFECV	Binary	24: CREA, Age, hypertension, PROTEIN, BUN, ALB, ALP, ALT, CA, P, TBIL, U.A, K, Mg, HDL, LDL, TRIGLYCERIDE, HCT, HGB, RBC, RDW-CV, PROTEINUrine, RBCUrine, WBCUrine
	Stage	7: CREA, BUN, P, HCT, HGB, RBC, PROTEINUrine
XGBoost		
RFE	Binary	28: CREA, Gender, Age, PROTEIN, BUN, ALP, ALT, AST, CA, CHOL, NA, P, TBIL, U.A, K, Mg, HDL, LDL, CL, TRIGLYCERIDE, HGB, MCHC, MCV, PLATEL, RDW-CV, PROTEINUrine, RBCUrine, WBCUrine
	Stage	18: CREA, Gender, Age, BUN, ALB, ALP, CA, CHOL, NA, TBIL, U.A, K, LDL, HCT, PLATEL, RBC, RDW-CV, RBCUrine
RFECV	Binary	10: CREA, Gender, Age, PROTEIN, BUN, ALP, ALT, NA, Mg, WBCUrine
	Stage	10: CREA (mg/dL), Gender, Age, BUN, ALB, TBIL, U.A, HCT, RBC, RBCUrine
AdaBoost		
RFE	Binary	15: CREA, Gender, Age, PROTEIN, NA, P, Mg, HDL, LDL, TRIGLYCERIDE, MCHC, PLATEL, WBC, PROTEINUrine, WBCUrine
	Stage	24: CREA, Gender, Age, PROTEIN, BUN, ALB, ALP, ALT, AST, GLU GLUCOSE, P, U.A, K, Mg, CL, TRIGLYCERIDE, HCT, HGB, MCH, MCV, RBC, RDW-CV, RBCUrine, WBCUrine
RFECV	Binary	26: CREA, Gender, Age, PROTEIN, BUN, ALB, ALP, ALT, AST, NA, P, TBIL, U.A, K, Mg, HDL, LDL, CL, TRIGLYCERIDE, HGB, MCHC, MCV, PLATEL, WBC, PROTEINUrine, WBCUrine
	Stage	10: CREA, Gender, Age, BUN, ALB, ALT, GLU GLUCOSE, HCT, MCV, RDW-CV
MLP		
SelectKBest	Binary	18: CREA, Age, hypertension, diabetes mellitus, BUN, ALB, ALP, CHOL, P, U.A, K, HDL, HCT, HGB, RBC, RDW-CV, PROTEINUrine, WBCUrine
	Stage	22: CREA, Gender, Age, PROTEIN, BUN, ALB, ALP, CA, NA, P, U.A, HDL, LDL, CL, HCT, HGB, RBC, WBC, RDW-CV, PROTEINUrine, RBCUrine, WBCUrine

**Table 6:** External validation results on the UCI CKD dataset for the binary classification component of the proposed framework, including hold-out and 5-fold cross-validation performance using the optimal number of selected features (k = 8).

Evaluation	K	Accuracy	Precision (Macro)	Recall (Macro)	F1-Score (Macro)	AUC
Hold-out (70/15/15)	8	0.983	1.000	0.973	0.986	1.000
5-Fold CV (mean ± std)	8	0.987 ± 0.009	0.984 ± 0.016	0.996 ± 0.009	0.990 ± 0.007	0.999 ± 0.000

**Table 7:** Binary head model comparison.

Model	End-to-End Runtime	# Flagged Non-CKD Cases
Random Forest + RFE	33 s	42
XGBoost + RFE	8 s	15
XGBoost + RFECV	19 s	17
MLP + SelectKBest	~2 min	12

**Table 8:** Representative examples of data-driven CKD-like cutpoints.

Feature	Non-CKD Range (Q5–Q95)	CKD-Like Cutpoint
CREA (mg/dL)	[0.545, 1.275]	≥1.63
BUN (mg/dL)	[7, 23.4899]	≥23.7
HGB	[10.1, 16.2]	≤10.1
HCT	[32, 48.75]	≤32

## Data Availability

The datasets presented in this article are not readily available due to privacy and ethical restrictions.

## Supplementary Materials

The following supporting information can be downloaded at: https://www.mdpi.com/article/10.3390/diagnostics16081157/s1. Table S1. Percentage of missing values for each feature computed from the original dataset prior to preprocessing.

## Appendix A

To ensure transparency, reproducibility, and clinical interpretability, the complete set of data-driven CKD-like cutpoints derived from the training cohort is provided in Table A1. These cutpoints were computed by analyzing the empirical distributions of each laboratory feature in both non-CKD and CKD populations. For each feature, the lower (Q5) and upper (Q95) quantiles of the non-CKD distribution were used to define cohort-specific reference ranges, while the CKD-like threshold was selected in the disease-associated direction. This approach enables identification of laboratory values that shift toward CKD-specific patterns rather than relying on conventional clinical reference intervals. The thresholds reported in Table A1 are intended exclusively for model interpretability and decision support within the proposed framework and are not designed to replace standard clinical laboratory cutoffs.

**Table A1:** Complete CKD-like cutpoints for all laboratory features.

Feature	Non-CKD Range (Q5–Q95)	CKD-Like Cutpoint
ALB (g/dL)	[3.1557, 4.7]	<=3.1557
ALP (U/L)	[47, 110.332]	>=110.332
ALT (U/L)	[9.2, 57.1]	<=9.2
AST (U/L)	[11.1, 42.0702]	<=11.1
Age	[24, 73.5]	>=73.5
BUN (mg/dL)	[7, 23.4899]	>=23.7
CA (mg/dL)	[6.94482, 12.3593]	<=6.94482
CHOL (mg/dL)	[120, 254.45]	<=120
CL	[97.0513, 107]	>=107
CREA (mg/dL)	[0.545, 1.275]	>=1.63
GLU GLUCOSE (mg/dL)	[74.3531, 232.235]	>=232.235
HCT	[32, 48.75]	<=32
HDL	[33.8108, 70.05]	<=33.8108
HGB	[10.1, 16.2]	<=10.1
K (MMOL/L)	[3.68, 4.885]	>=4.885
LDL	[53.9651, 170.228]	<=53.9651
MCH	[22.6, 31.45]	>=31.45
MCHC	[30.6, 34.8]	>=34.8
MCV	[71.45, 95.35]	<=71.45
Mg (mg/dL)	[1.67, 2.13]	>=2.13
NA (MMOL/L)	[132, 142]	>=142
P (mg/dL)	[2.675, 4.3]	>=4.3
PLATEL	[165, 443.5]	<=165
PROTEIN (g/dL)	[6.65263, 8.00493]	<=6.65263
RBC	[3.79, 5.815]	<=3.79
RBCUrine	[0.1, 10.1429]	>=10.1429
RDW-CV	[12.2, 17.05]	>=17.05
TBIL (mg/dL)	[0.0606559, 0.902033]	<=0.0606559
TRIGLYCERIDE	[54.65, 219.256]	>=219.256
U.A (mg/dL)	[3.2, 7.65559]	>=7.65559
WBC	[3.78, 10.53]	>=10.53
WBCUrine	[0, 42.1791]	>=42.1791
